# Chiral nanoparticles in singular light fields

**DOI:** 10.1038/srep45925

**Published:** 2017-04-05

**Authors:** Ilia A. Vovk, Anvar S. Baimuratov, Weiren Zhu, Alexey G. Shalkovskiy, Alexander V. Baranov, Anatoly V. Fedorov, Ivan D. Rukhlenko

**Affiliations:** 1 Center of Information Optical Technologies, ITMO University, Saint Petersburg 197101, Russia; 2 Department of Electronic Engineering, Shanghai Jiao Tong University, Shanghai 200240, China; 3 Monash University, Clayton Campus, Victoria 3800, Australia; 4 Saint Petersburg State University, 7–9 University Embankment, Saint Petersburg 199034, Russia; 5 Institute for Design Problems in Microelectronics of Russian Academy of Sciences, Moscow 124365, Russia

## Abstract

The studying of how twisted light interacts with chiral matter on the nanoscale is paramount for tackling the challenging task of optomechanical separation of nanoparticle enantiomers, whose solution can revolutionize the entire pharmaceutical industry. Here we calculate optical forces and torques exerted on chiral nanoparticles by Laguerre–Gaussian beams carrying a topological charge. We show that regardless of the beam polarization, the nanoparticles are exposed to both chiral and achiral forces with nonzero reactive and dissipative components. Longitudinally polarized beams are found to produce chirality densities that can be 10^9^ times higher than those of transversely polarized beams and that are comparable to the chirality densities of beams polarized circularly. Our results and analytical expressions prove useful in designing new strategies for mechanical separation of chiral nanoobjects with the help of highly focussed beams.

The current progress of pharmaceutical research and technology is slowed down by the absence of a unified method of separation of enentiometic forms of chiral drug molecules, which often exhibit profoundly different interactions with biological tissues^1^. The separation of molecular enantiomers is an extremely difficult task, because they are indistinguishable through interactions with achiral objects and have mostly identical physical properties, such as densities, solubilities, boiling and melting points, etc.^2^. Chiral chromatography, derivatization, and other well developed techniques of racemate resolution are useful on analytical scale^3^,^4^, but become highly inefficient and quite expensive where kilograms of different chiral mixtures need to be quickly and reliably purified^5^.

A promising approach to tackling this problem is in using enantioselective mechanical forces induced by chiral light^6^,^7^,^8^,^9^,^10^. Unfortunately, the interaction of circular polarized light with chiral molecules themselves is naturally weak due to the smallness of molecules compared to the optical wavelength and because of the relatively small number of atoms constituting them^11^,^12^. A much stronger chiroptical response is featured by chiral plasmonic complexes^13^,^14^, chiral semiconductor nanocrystals^15^,^16^,^17^,^18^,^19^,^20^,^21^,^22^,^23^, quantum-dot molecules^24^, helix-type quantum-dot supercrystals^25^, and ‘Swiss-roll’ structures^26^. While all of them can in principle be used to advance existing and create new techniques for sensing, separation, and delivery of enantiomeric drug molecules, semiconductor nanocrystals appear to be especially promising due to their resistance to photobleaching, tuneable energy spectrum, and highly selective interaction with both enantiomeric molecules and living tissues^27^,^28^.

Our recent study of interaction of chiral nanoparticles with circularly polarized light has revealed an optimal field pattern which eliminates the achiral forces acting on the nanoparticles and maximizes the chiral ones^29^. This paper continues our research on the interaction of nanostructured chiral matter with chiral light fields. We use the dipolar approximation to analytically calculate optical forces that are exerted on small chiral nanoparticles by the electromagnetic fields of Laguerre-Gaussian beams of different polarizations. Since the chiral part of the force is induced by chirality flow and the gradient of chirality density, these quantities are also treated analytically and thoroughly analysed. We hope that this study will be useful in the development of new enantioseparation techniques and stimulate new experiments.

A small chiral nanoparticle can be modelled as a point dipole of electric polarizability 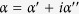 and mixed electric-magnetic polarizability 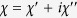. Suppose that such a dipole is exposed to the field of a laser beam of frequency *ω* propagating in the positive direction of the *z* axis. To study the chiral effects associated with the inhomogeneity of the beam in the transverse plane, we go beyond the scalar approximation and define the electromagnetic field of the beam through its vector potential 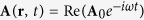, where


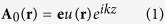


is the time-independent phasor, **e** is the unit polarization vector, *k* = *ω*/*c* is the wave number, and *u*(**r**) the complex amplitude of the beam. In what follows, we restrict ourselves to considering Laguerre-Gaussian beams, whose amplitude is given by


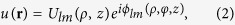


where


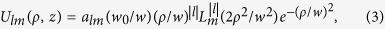



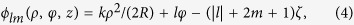


*a*_*lm*_ is the normalization constant, 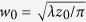 is the waist radius, 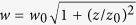 is the beam width, 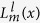 is the associated Laguerre polynomial, 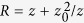 is the wavefront curvature radius, 
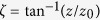
 is the phase delay, *l* = 0, ±1, ±2, … is the topological charge of the beam, and *m* = 0, 1, 2, … is the number of nodes in the radial direction.

The electric and magnetic fields of the beam, defined similar to the vector potential as 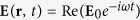 and 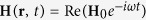, are given in the Lorenz gauge by the phasors


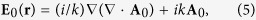



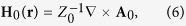


where 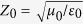 is the impedance of free space. By substituting Eq. (1) into Eqs (5) and (6) and using the paraxial approximation 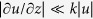, we arrive at the following general expressions for the electric and magnetic fields:


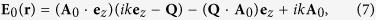



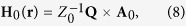


where


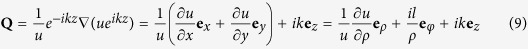


and we have taken into account that 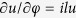.

The fields are normalized to the total power carried by the beam, which is given by the *z* component of its Poynting vector 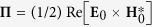 integrated over the entire transverse plane,


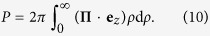


The time-averaged forces and torques acting on a chiral nanoparticle are given by^30^,^31^


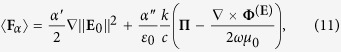



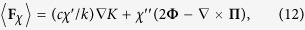



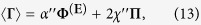


where 
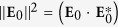
, 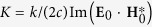 is the chirality density or simply ‘chirality’, 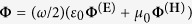 is the chirality flow, and 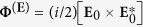 and 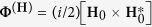 are the electric and magnetic ellipticities. The signs of 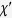 and 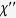 distinguish between the two nanoparticle enantiomers and determine the directions of the chiral force and torque. In what follows, we shall focus on 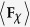 and 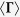 while assuming that the achiral force is cancelled or minimized by the off-resonant excitation of nanoparticles^31^.

The chiral optical force produced by a Laguerre-Gaussian beam can be used to separate chiral nanoparticles exhibiting sufficiently strong circular dichroism (CD). The CD signal is proportional to the imaginary part of the electric-magnetic polarizability, 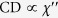, and determines the degree of enantioselectivity in the nanoparticle interaction with chiral light^29^. Of course, in addition to pure electric and electric-magnetic polarizabilities, nanoparticles also possess a much smaller magnetic polarizability, 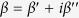, which is ignored in Eqs (11,12,13). Since the three polarizabilities of a nanoparticle of size *a* are related as the three consecutive powers of *ka* — 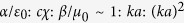 — the assumption of zero magnetic polarizability is well justified as long as *ka* ≪ 1. But even if the nanoparticle is not small compared to the optical wavelength, its pure magnetic response contributes to the achiral component of the optical force and does not affect the chiral one^31^.

## Results and Discussion

We begin by analysing how a chiral dipole interacts with a light beam produced by a transversely polarised vector potential. By setting **e** = **e**_x_ in Eqs (7) and (8), we find that the electric and magnetic fields of the beam are given by


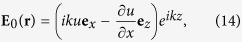



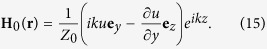


These fields are almost completely transverse, with small longitudinal components originating from the dependence of *u* on the transverse coordinates.

Using the obtained fields in the above definitions yields


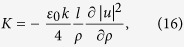



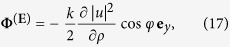



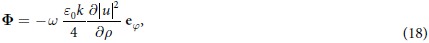



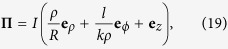


where 
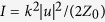
 is the local intensity of the beam. Equations (10) and (19) give the normalization constant of the form


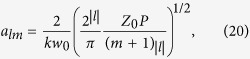


where 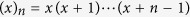 for *n* = 1, 2, 3, … and (*x*)_0_ = 1 is the Pochhammer symbol^32^.

One can see that a chiral dipole illuminated by a transversely polarized Laguerre-Gaussian beam carrying a nonzero orbital angular momentum 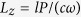 exhibits both achiral and chiral forces, each of which has a reactive and dissipative components. Since the characteristic *ρ*- and *z*-scales of chirality variations are *w* and z_0_, and *z*_0_ ≫ *w*, the reactive chiral force has a dominant *ρ* component and the dissipative chiral force has a dominant *z* component, which are about *z*_0_/*w* times larger than the nondominant ones:


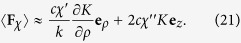


**Figure 1:**
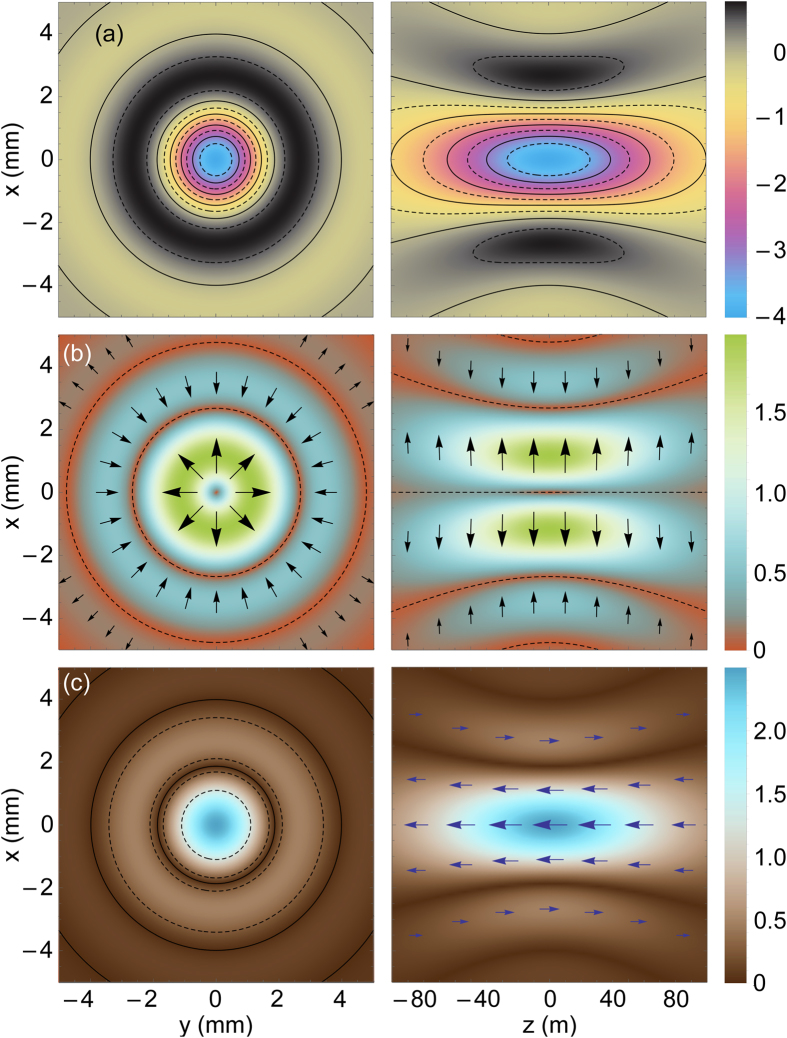
(**a**) Normalized chirality density, (**b**) reactive component of chiral force, and (**c**) dissipative component of chiral force in planes *z* = 0 (left panels) and *y* = 0 (right panels) for transversely polarized Laguerre-Gaussian beam with *l* = *m* = 1, *λ* = 500 nm, and *z*_0_ = 100 m. The chirality and the magnitudes of forces on the density plots are in the units of 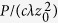, 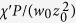, and 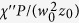.

Figure 1 shows chirality density, reactive part of the chiral force, and dissipative part of the chiral force produced by the transversely polarized Laguerre-Gaussian beam with *l* = *m* = 1. The sign of chirality near the center of this beam and the beams with *l* > 0 coincides with the sign of chirality of right circularly polarized light. For *l* = ±1 the chirality on the beam axis scales like ∝ *m* + 1. Panels (a) and (b) of the figure illustrate the fact that the dissipative part of chiral force, given by the second term in Eq. (21), is fully determined by the chirality density. For *l* = 1 this density peaks at the beam axis, where the optical intensity is zero. For larger *l* the central peak disappears, because the first derivative of intensity with respect to the radius vanishes at *ρ* = 0, and the transverse distribution of chirality density has a ring structure. In this case, the maximal chirality resides in the first (closest to the axis) ring, near the point where function *I*(*ρ*) has an inflection. On the other hand, the reactive part of the chiral force scales in proportion to the first derivative of chirality density. Panel (c) shows that, depending on the sign of 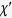, chiral nanoparticles are either pulled into the area of negative chirality near the beam axis or pulled out of it.

Another beam of practical interest is the one with a circularly polarized vector potential. Its electric and magnetic fields are


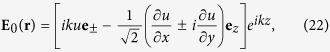



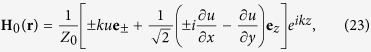


where 
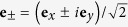
 and the plus or minus sign corresponds to, respectively, the left circularly polarized (LCP) or right circularly polarized (RCP) vector potential. Similar to the previous case, the field polarization nearly coincides with the polarization of **A**_0_.

The fields of circularly polarized beam (CPB) yield the following chirality density, chirality flow, and Poynting vector:


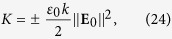



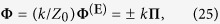



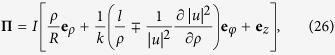


where


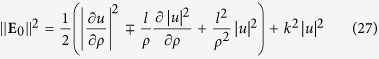


and the normalization constant is given by Eq. (20).

**Figure 2:**
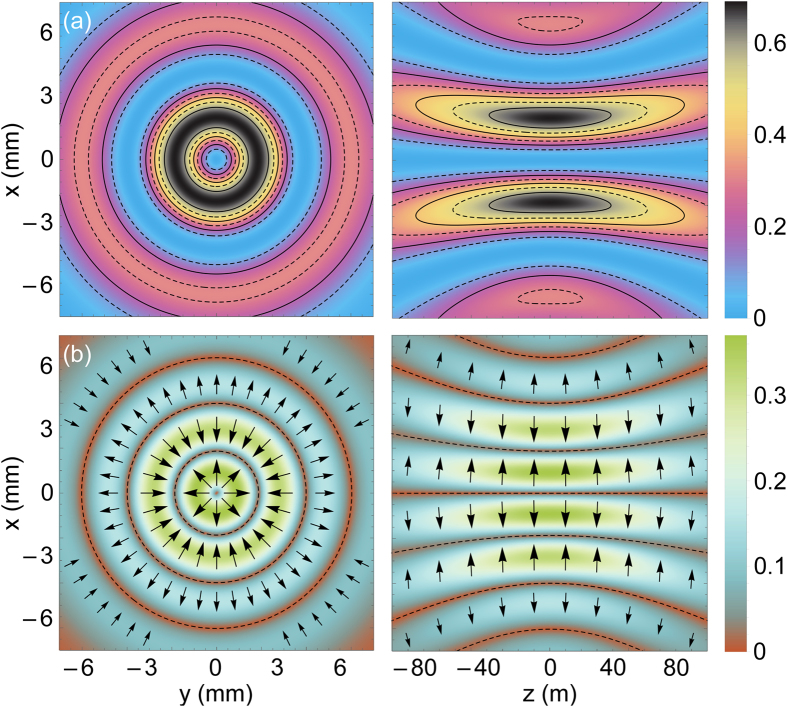
The same as in Fig. 1(a) and (b) but for left circularly polarized Laguerre-Gaussian beam with *l* = *m* = 1; the chirality and the magnitude of the force are in the units of 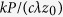 and 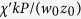.

Much like the transversely polarized beam (TPB) of linear polarization, the CPB carries angular momentum *L*_*z*_ = *lP*/(*cω*) and exerts on a chiral dipole two kinds of reactive and dissipative forces. In contrast to the linearly polarized beam, the chirality flow of the CPB exceeds the curl of the Poynting vector throughout the entire space except the immediate vicinity of the phase singularity. As a consequence, the dissipative part of the chiral force is, again, proportional to chirality, 
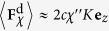
. Figure 2 shows chirality density and reactive part of chiral force produced by the CPB with *l* = *m* = 1. The distribution of chirality density follows the intensity profile of the beam and is nonzero even in the absence of topological charge. The characteristic radius of this distribution is larger than in the case of TPB, which is evidenced by the comparison of Fig. 2(a) with Fig. 1(a) and Eq. (24) with Eq. (16). The radial component of the reactive chiral force is proportional to the derivative 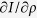 of beam intensity and, therefore, has a complex ring structure even for small *m*. At the same time, the magnitude of this force decays with the distance from the beam axis more gradually than in the case of TPB.

Finally, we consider a Laguerre-Gaussian beam with a longitudinally polarized vector potential (**e** = **e**_*z*_), which does not have a plane-wave analogue. The electric and magnetic fields of this beam,


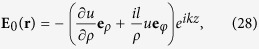



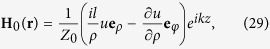


both vanish for purely transverse plane waves, with *u* = const and *l* = 0. With these fields, the above definitions give


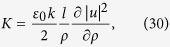



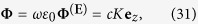



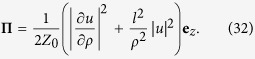


Some algebra leads to the normalization constant


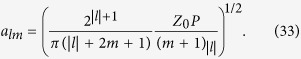


In contrast to the previous two cases, the longitudinally polarized beam (LPB) does not carry an angular momentum in the *z* direction, because 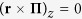. But similar to the CPB, for 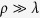 we have 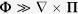 and 
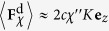
. The chirality density produced by a LPB is similar in form to Eq. (16). However, due to different intensities of the two beams, their actual chiralities can differ by many orders of magnitude. This can be seen from Eqs (16), (20), (24), (30), and (33), which give the following ratios of chirality density of LPB to the chiralities of TPB and CPB far away from the phase singularity:


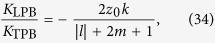



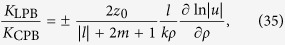


where |*u*| is the profile of either LPB or CPB, and we have taken into account that for CPB 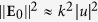. It is easy to see that 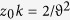, where 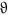 is the angular radius of cone which is asymptotically approached by the 1/*e*^2^ irradiance contours of the beam. Hence, one can enhance the chirality of LPB as compared to the chirality of TPB by simply reducing the divergence of the beam. In the above examples, with *z*_0_ = 100 m and *λ* = 500 nm, the enhancement factor exceeds 10^9^. By further estimating near *z* = 0 that *ρ* ~ *w*_0_ and 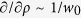, we find from Eq. (35) that 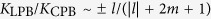. This result implies that the LPBs with *m* = 0 can produce chiralities that are comparable in strength to the chirality of circularly polarized Gaussian beam of zero topological charge.

**Figure 3:**
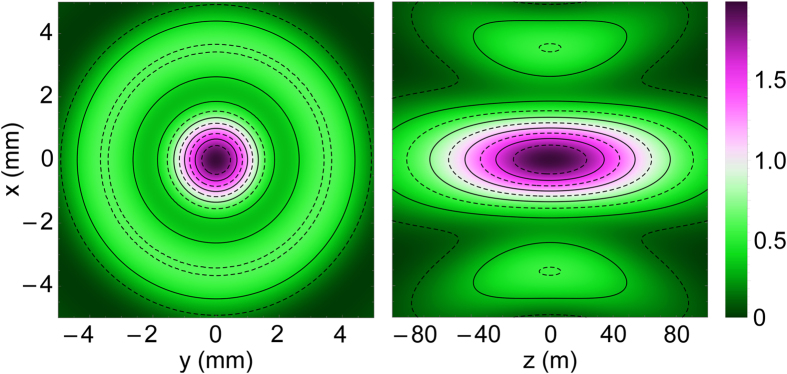
Magnitude of Poynting vector of longitudinally polarized Laguerre-Gaussian beam with *l* = *m* = 1; the unit of measurement is 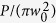.

The *z* component of the Poynting vector of the LPB with *l* = *m* = 1 is shown in Fig. 3. An interesting feature to note here is that the Poynting vector reaches its maximal value on the beam axis. This feature originates from the derivative in Eq. (32) and is specific to LPBs with *l* = ±1. Indeed, for *ρ* → 0 one can show that


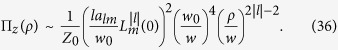


By considering here that 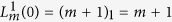, for *l* = ±1 we get 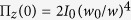, where 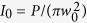.

As a concluding remark, we would like to note the need of a tradeoff between the magnitude of the enantioselective optical forces exerted on chiral nanoparticles by TPB and LPB and the degree of spatial separation provided by them. This conclusion simply follows from the fact that these forces are determined by the derivatives of the beam intensity and, therefore, are the stronger the smaller the typical length scale of intensity variations. Hence, an increase of the beam waste *w*_0_ or a reduction of the number of radial nodes *m* for a given beam power inevitably reduces the chiral optical forces.

In conclusion, we have calculated optical forces and torques exerted on chiral nanoparticles by Laguerre-Gaussian beams of transverse, circular, and longitudinal polarizations. The chirality density of the longitudinally polarized beam was found to be comparable to the chirality density of the circularly polarized beam. For a weakly divergent beam, it can exceed the chirality of the transversely polarized beam by a factor of 10^9^. It was also shown that regardless of their polarization, Laguerre-Gaussian beams with topological charges exert on the nanoparticles chiral and achiral forces with both reactive and dissipative components. We believe that our findings and derived analytical expressions will help to solve the problem of optomechanical separation of nanoparticle enantiomers.

## Additional Information

**How to cite this article:** Vovk, I. A. *et al*. Chiral nanoparticles in singular light fields. *Sci. Rep.* **7**, 45925; doi: 10.1038/srep45925 (2017).

**Publisher's note:** Springer Nature remains neutral with regard to jurisdictional claims in published maps and institutional affiliations.
